# Extra-Soft Tactile Sensor for Sensitive Force/Displacement Measurement with High Linearity Based on a Uniform Strength Beam

**DOI:** 10.3390/ma14071743

**Published:** 2021-04-01

**Authors:** Na Ni, Xiaomin Xue, Dongbo Li

**Affiliations:** 1 School of Science, Xi’an University of Architecture and Technology, Xi’an 710055, China; nina@xauat.edu.cn; 2 Department of Civil Engineering, Xi’an Jiaotong University, Xi’an 710054, China

**Keywords:** displacement sensor, force sensor, ionic skin, uniform strength beam

## Abstract

The soft sensing system has drawn huge enthusiasm for the application of soft robots and healthcare recently. Most of them possess thin-film structures that are beneficial to monitoring strain and pressure, but are unfavorable for measuring normal displacement with high linearity. Here we propose soft tactile sensors based on uniform-strength cantilever beams that can be utilized to measure the normal displacement and force of soft objects simultaneously. First, the theoretical model of the sensors is constructed, on the basis of which, the sensors are fabricated for testing their sensing characteristics. Next, the test results validate the constructed model, and demonstrate that the sensors can measure the force as well as the displacement. Besides, the self-fabricated sensor can have such prominent superiorities as follows—it is ultra-soft, and its equivalent stiffness is only 0.31 N·m^−1^ (approximately 0.4% of fat); it has prominent sensing performance with excellent linearity (R^2^ = 0.999), high sensitivity of 0.533 pF·mm^−1^ and 1.66 pF·mN^−1^ for measuring displacement and force; its detection limit is as low as 70 μm and 20 μN that is only one-tenth of the touch of a female fingertip. The presented sensor highlights a new idea for measuring the force and displacement of the soft objects with broad application prospects in mechanical and medical fields.

## 1. Introduction

Nowadays, soft tactile sensors have received increasing attention because they can be widely applied to the assistive soft robot, healthcare and entertainment fields [1,2,3]. In contrast to traditional rigid sensors, advantageous compliant properties of soft sensors render them safe to monitor gentle movements or interactions with humans [4,5]. They have promising applications in health monitoring systems, electronic skin, and wearable devices [2,3,4].

Given the demand, different transduction methods of flexible tactile sensors have been developed, such as piezoresistance [6,7], capacitance [8,9], piezoelectricity [10,11], triboelectricity [12,13], and optics [14]. Among these different kinds of flexible tactile sensors, capacitance sensors show better potential in terms of static measurement, high sensitivity, and economical fabrication processes [15,16]. These capacitive tactile sensors mainly comprise dielectric elastomers and flexible electrodes. They can be used to measure strain, pressure, and three-axis force by converting mechanical variations into capacitance signals. For instance, the dielectric elastomers (dielectric layers) sandwiched between two-set compliant conductors are fabricated for strain and pressure sensors [17,18,19]. Microstructure rubber dielectric layers are developed for highly sensitive pressure sensors [8,20,21]. The dielectric layer with compressible dielectric and air gaps is adopted for normal force [22] and three-axis force sensors [15,23]. Although these sensors show good performance towards artificial touch, they are not suitable for measuring the displacement or concentrated force perpendicular to an object surface (i.e., normal displacement or force) due to their thin film forms.

In view of that, the beam-like structures are developed that can be used to measure the normal displacement or normal force [24,25]. However, most of them are rigid and unsuitable for applications in soft devices and health monitoring [25,26,27]. Only a few have reported soft displacement or force sensors based on soft beams integrated with flexible sensing elements. Lucarotti et al. [24] reported a method to sense force and deflection in a soft body based on beam configuration, with the minimum detectable force of 10 mN. Kanao et al. [28] proposed a tactile sensor based on a printed strain sensor on a soft beam, which can be used to detect the minimum tactile force of 20 mN. Lee et al. [29] fabricated a soft cantilever-structured capacitance sensor, which yields a sensitivity of 0.006% kPa^−1^. Kim et al. [30] developed a crack sensor integrated with a silicon rubber beam for the measurement of cardiac contractility by measuring deflection of the cantilever. Those investigations have significantly advanced the development of soft force/displacement sensors, but cannot achieve high sensitivity, and especially excellent linearity for high precision detection. This is because those beams all have uniform cross-section, which is disadvantageous to homogeneous deformation of the sensing elements on the beam.

To measure the normal force and displacement with high linearity and sensitivity, this paper set out to propose an extremely soft tactile sensor based on a uniform-strength cantilever beam. The sensor consists of a capacitive sensing sheet and a uniform strength beam that are attached altogether. The normal force/displacement applied at the free end of the beam can be measured according to the capacitance change of the capacitive sensing sheet. It is worth mentioning that the uniform strength beam structure guarantees uniform strain along the beam length, resulting in the homogeneous deformation of sensing sheets. Consequently, there is a highly linear relationship between the capacitance change and the applied force/displacement, ensuring the proposed sensor has excellent linearity.

The research contents are as follows—first, the theoretical model is derived, that can describe the relationship between the force/displacement (input) and the capacitance change (output). Secondly, three sensor samples are fabricated based on ionic conductors and dielectric elastomers in pursuit of ultra-softness. Next, their sensing performances are observed through experiments. As verified, the results from the theoretical model are in good agreement with the experiments. Finally, the minimum detection force and equivalent stiffness of the proposed sensor are tested so as to demonstrate their superiorities like sensitivity and softness. In summary, the experimental results successfully demonstrate that the proposed sensor not only has excellent linearity and sensitivity but also extremely low equivalent stiffness. Therefore, the sensor is well suitable for measuring the normal displacement/force of soft objects, exhibiting its potential in the applications of soft machines, human monitoring and so on.

## 2. Sensor Design and Theoretical Model

### 2.1. Sensor Structure

**Figure 1:**
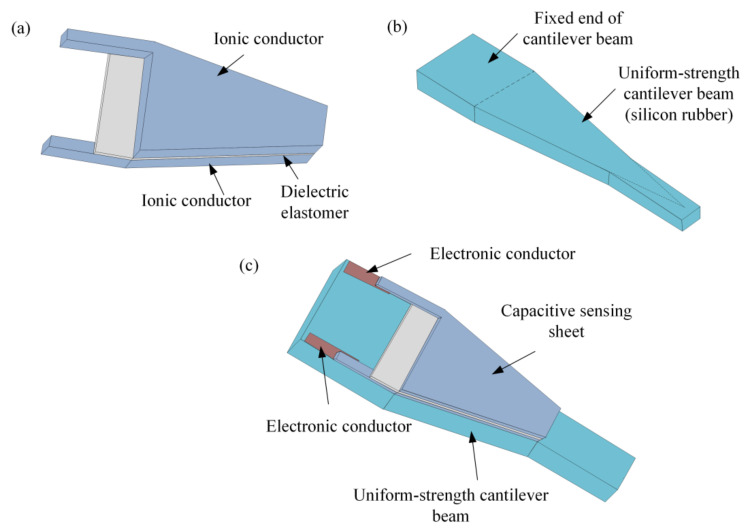
Schematic diagram of the tactile sensor where (**a**) is the capacitive sensing sheet, (**b**) is the uniform- strength cantilever beam, and (**c**) is the tactile sensor by assembly of the capacitive sensing sheet to the uniform-strength cantilever beam.

We developed a soft tactile force/displacement sensor, constituting a uniform- strength cantilever beam and capacitive sensing sheet. The capacitive sensing sheet is designed such that the dielectric layer (Eco-Flex 00-50) is sandwiched between two ionic conductors, as shown in Figure 1a. Figure 1b shows the structure of the soft uniform-strength cantilever beam (silicon rubber). The capacitive sensing sheet is attached to the soft cantilever beam with the viscidity of ionic conductors (hydrogels), and a thin line of each ionic conductor is connected to a metallic electrode (electronic conductor) fastened onto the fixed end of the cantilever beam, which creates a sensing system (see Figure 1c).

### 2.2. Sensing Principle

**Figure 2:**
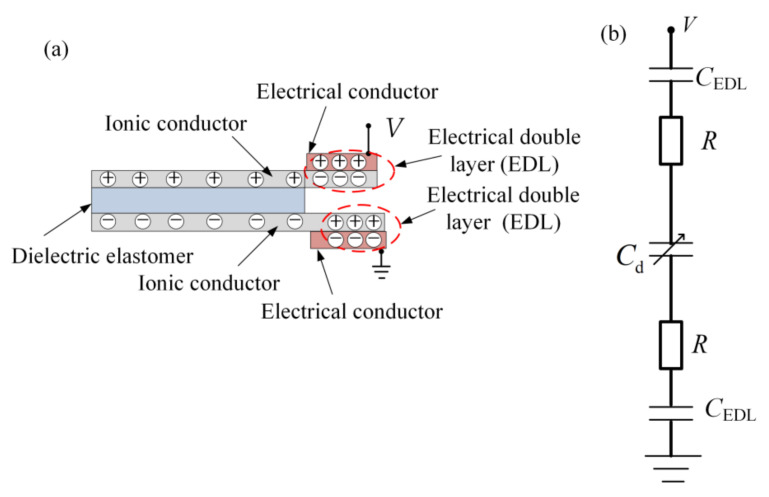
(**a**) Charge distribution and (**b**) equivalent circuit of ionic skin [17].

The capacitive sensing sheet constituting one dielectric and two ionic conductors is called ionic skin. First, the strain sensing principle of the ionic skin is described as follows [17]—it is formed a hybrid ionic-electronic circuit by connecting the ionic conductors to electronic conductors for measuring capacitance value (see Figure 2). When low voltage is applied between the two electronic conductors, as shown in Figure 2a, each electrical double layer forms at the interface between the electronic conductor and the ionic conductor, similar to a capacitor (*C*_EDL_). Simultaneously, the capacitor of the dielectric is formed using ionic conductors and the dielectric. As shown in Figure 2b, the capacitors of the electrical double layers (*C*_EDL_) are in series with the capacitor of the dielectric ($C_{d}$). The measured capacitance (*C*) between the two electronic conductors can be given as $C=C_{d}/(2C_{d}/C_{\mathrm{EDL}}+1)$. The capacitance of the electrical double layer is considerably higher than that of the dielectric, i.e., $C_{d}/C_{\mathrm{EDL}}\approx 0$, because the separated distance (nanometers) of charges in the electronic conductor and in the ionic conductor is substantially lower than that of charges separated through the dielectric, whose thickness is >0.01 mm. Consequently, the dielectric capacitance ($C_{d}$) dominates the measured capacitance, i.e., $C\approx C_{d}$. When the capacitive sensing sheet is uniaxially stretched λ times, the capacitance change (ΔC) induced by the uniaxial tensile strain ($\varepsilon_{d}$) of the capacitive sensing sheet can be given as follows:

(1) $$\Delta C=C_{0}\varepsilon_{d}.$$

where $C_{0}$ is the capacitance of the capacitive sensing sheet in the undeformed state.

**Figure 3:**
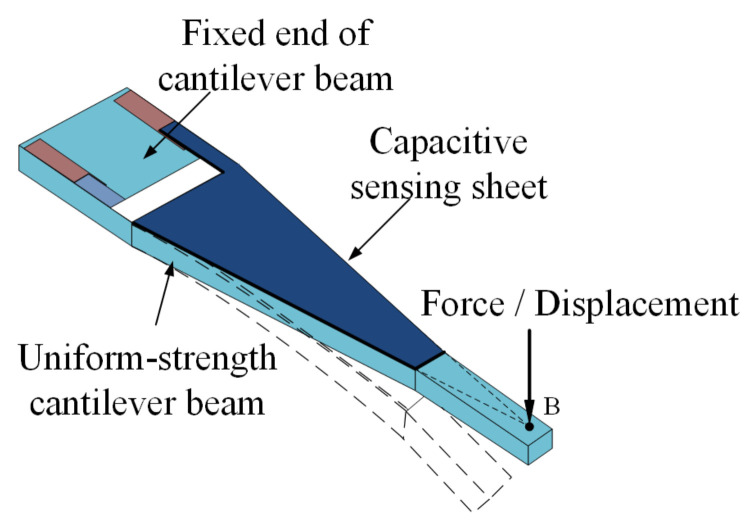
Deformation of the tactile sensor induced by normal force/displacement at the free end of the beam.

When normal force or displacement is applied at point B of the free extremity, as shown in Figure 3, it deforms the uniform-strength cantilever beam and capacitive sensing sheet. The deformation of the capacitive sensing sheet leads to a capacitance change according to the aforementioned Equation (1). Therefore, the tactile sensor can be used to detect the normal force/displacement by measuring the capacitance change.

### 2.3. Theoretical Model

**Figure 4:**
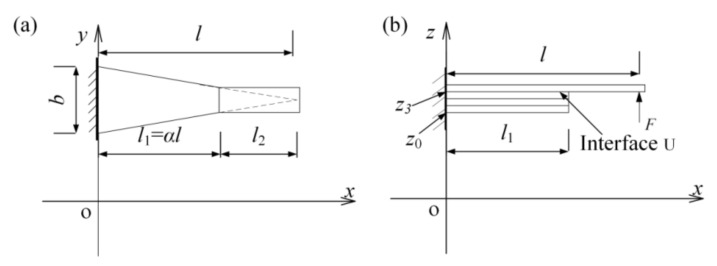
Geometry of the tactile sensor in (**a**) top view and (**b**) side view.

When normal force/displacement is applied at the free end of the cantilever sensor (see Figure 3), the correlation between the capacitance change and normal force/displacement is derived. The geometry of the sensor is shown in Figure 4. The sensor includes two segments (*l*_1_ and *l*_2_) in the x-direction. Different segments comprise a different number (*k*) of layers. For the segment 1, *k* = 4, in which one is beam and three are the capacitance-sensing sheet. For the segment 2, *k* = 1, that is the beam itself. For deriving a reasonably simplified model, some basic assumptions should be set as—(1) the sensor is in a static equilibrium and small deformation state; (2) the Euler–Bernoulli model is employed here, because the uniform-strength cantilever beam is relatively long; and (3) adjacent layers are all compatible, adjacent segments of the beam are continuous.

According to the derivation of the deflection of the multimorph beam [31], the strain of the capacitive sensing sheet induced by the force at the free end of the uniform strength beam is derived as follows. For the segment *j* (*j* = 1 or *j* = 2), the total axial force (the *k*-layer beam) at any cross section in equilibrium state can be given as:

(2) $$\sum_{i=1}^{k}P_{ij}=0,$$

where $P_{ij}$ is the axial force at any cross section of the element of the layer *i* and segment *j*. The axial force is equal to an integral over all normal stress at each cross section. The total axial force is given by:

(3) $$\sum_{i=1}^{k}P_{ij}=b(x)\sum_{i=1}^{k}\int_{z_{i-1}}^{z_{i}}E_{ij}\times \varepsilon_{ij}dz=0,$$

where b(x) is the width of the beam at any cross section, $E_{ij}$ is the elastic modulus of the element (the layer *i*, segment *j*), and $\varepsilon_{ij}$ is the strain (x-direction) of the element. In Figure 4, the length of the capacitive sensing sheet $l_{1}$ is equal to αl, where α is the ratio of the length of the capacitive sensing sheet and the cantilever beam. Thus, b(x) can be expressed as:

(4) $$b(x)=\{\begin{array}{cc} \frac{b(l-x)}{l}\;j=1\;(0\leq x\leq l_{1}) & \left(a\right)\\ \left(1-\alpha \right)b\;\;j=2\;\;(l_{1}\leq x\leq l) & \left(b\right) \end{array}$$

Adjacent layers are all compatible, $\varepsilon_{ij}$ can be given as:

(5) $$\varepsilon_{ij}=(z_{Nj}-z)\frac{\partial^{2}w_{j}}{\partial x^{2}},$$

where $z_{Nj}$ is the position of the neutral axis of the segment *j*, $w_{j}$ is the deflection of the segment *j*. To substitute Equation (5) into Equation (3), $z_{Nj}$ can be expressed as:

(6) $$z_{Nj}=\frac{\sum_{i=1}^{k}E_{ij}h_{i}^{2}}{\sum_{i=1}^{k}E_{ij}h_{i}},$$

where $h_{i}=z_{i}-z_{i-1}$, $h_{i}{}^{2}=(z_{i}{}^{2}-z_{i-1}{}^{2})/2$. The internal stresses produced internal moments. The internal moments are balanced with external moments at any axial position, i.e.,

(7) $$F(l-x)+b(x)\sum_{i=1}^{k}\int_{z_{i-1}}^{z_{i}}T_{ij}zdz=0,$$

where F is the normal force applied to the free end of the beam, and $T_{ij}$ is the stress (x-direction) of the element. The stress can be expressed as follows:

(8) $$T_{ij}=E_{ij}\varepsilon_{ij}.$$

By substituting Equations (5) and (8) in Equation (7), the equation becomes:

(9) $$F(l-x)+b(x)(z_{Nj}\frac{\partial^{2}w_{j}}{\partial x^{2}}\sum_{i=1}^{k}E_{ij}h_{i}^{2}-\frac{\partial^{2}w_{j}}{\partial x^{2}}\sum_{i=1}^{k}E_{ij}h_{i}^{3})=0,$$

where $h_{i}{}^{3}=(z_{i}{}^{3}-z_{i-1}{}^{3})/3$.

By substituting Equations (4a), (6), and (9) in Equation (5), the strain along the x-direction of the element for the layer *i* and segment 1 can be written as

(10) $$\varepsilon_{i1}=(z_{N1}-z)\frac{Fl}{A_{1}b},$$

where $A_{1}=\sum_{i=1}^{4}E_{i1}h_{i}{}^{3}-{\left(\sum_{i=1}^{4}E_{i1}h_{i}{}^{2}\right)}^{2}/\sum_{i=1}^{4}E_{i1}h_{i}$. According to Equation (10), the following is the strain of the interface U (the coordinate point in the z direction is $z_{3}$) between the beam and capacitive sensing sheet

(11) $$\varepsilon_{U}=(z_{N1}-z_{3})\frac{Fl}{A_{1}b}.$$

Because the capacitive sensing sheet is glued to the beam, the strain of the capacitive sensing sheet is equal to that of the interface U, which leads to Equation (12)

(12) $$\varepsilon_{d}=\varepsilon_{U}.$$

By substituting Equations (11) and (12) in Equation (1), the capacitance change of the tactile sensor can be given as

(13) $$\Delta C=\frac{C_{0}l(z_{N1}-z_{3})}{A_{1}b}F.$$

Next, the deflection of the beam (multimorph cantilever beam) is derived as follows. By plugging Equation (6) into Equation (9), the differential equation for the deflection of the beam can be expressed as

(14) $$\frac{\partial^{2}w_{j}}{\partial x^{2}}=\frac{F(l-x)}{A_{j}b(x)}.$$

Defined $A_{j}$ as

(15) $$A_{j}=\sum_{i=1}^{k}E_{ij}h_{i}{}^{3}-{\left(\sum_{i=1}^{k}E_{ij}h_{i}{}^{2}\right)}^{2}/\sum_{i=1}^{k}E_{ij}h_{i}.$$

For segment 1, the differential equation is provided by substituting Equation (4a) into Equation (14), i.e.,

(16) $$\frac{\partial^{2}w_{1}}{\partial x^{2}}=\frac{Fl}{A_{1}b}.$$

The general solution to Equation (16) is

(17) $$w_{1}=\frac{Fl}{2A_{1}b}x^{2}+C_{11}x+C_{21},$$

where $C_{11}$ and $C_{21}$ are constants. Due to the boundary conditions of the beam, the deflection and slope are zero at clamped.

(18) $$w_{1}(0)=0,\;\frac{\partial w_{1}}{\partial x}=0.$$

According to Equations (17) and (18), the solved constants $C_{11}$ and $C_{21}$ are

(19) $$C_{11}=0,\;C_{21}=0.$$

By plugging Equation (19) into Equation (17), the deflection of segment 1 of the beam can be given as:

(20) $$w_{1}=\frac{Fl}{2A_{1}b}x^{2}.$$

For segment 2, the differential equation is provided by substituting Equation (4b) into Equation (14), i.e.,

(21) $$\frac{\partial^{2}w_{2}}{\partial x^{2}}=\frac{F(l-x)}{(1-\alpha )A_{2}b}.$$

The general solution to Equation (21) is:

(22) $$w_{2}=\frac{F\left(3lx^{2}-x^{3}\right)}{6(1-\alpha )A_{2}b}-\frac{5F}{12A_{2}b}+C_{12}x+C_{22}.$$

The deflection and slope of segment 1 are equal to those of segment 2 at x=αl.

(23) $$w_{1}\left|{}_{x=\alpha l}\right.=w_{2}\left|{}_{x=\alpha l}\right.,$$

(24) $$\frac{\partial w_{1}}{\partial x}\left|{}_{x=\alpha l}\right.=\frac{\partial w_{2}}{\partial x}\left|{}_{x=\alpha l}\right..$$

According to Equation (22), the constants $C_{12}$ and $C_{22}$ can be obtained by solving Equations (23) and (24).

(25) $$C_{12}=\frac{\alpha Fl^{2}}{A_{1}b}+\frac{\alpha \left(\alpha -2\right)Fl^{2}}{2\left(1-\alpha \right)A_{2}b},$$

(26) $$C_{22}=-\frac{\alpha^{2}Fl^{3}}{2A_{1}b}+\frac{\left(3\alpha^{2}-2\alpha^{3}\right)Fl^{3}}{6\left(1-\alpha \right)A_{2}b}.$$

By substituting Equations (25) and (26) into Equation (22), the deflection of segment 2 can be written as:

(27) $$w_{2}=\frac{F\left(3lx^{2}-x^{3}\right)}{6\left(1-\alpha \right)A_{2}b}+(\frac{\alpha Fl^{2}}{A_{1}b}+\frac{\alpha \left(\alpha -2\right)Fl^{2}}{2\left(1-\alpha \right)A_{2}b})x+\frac{\left(3\alpha^{2}-2\alpha^{3}\right)Fl^{3}}{6\left(1-\alpha \right)A_{2}b}-\frac{\alpha^{2}Fl^{3}}{2A_{1}b}.$$

Thus, the deflection of the beam at x=l can be written as:

(28) $$w_{2}(l)=\frac{\left(2\alpha -\alpha^{2}\right)Fl^{3}}{2A_{1}b}+\frac{{\left(1-\alpha \right)}^{2}Fl^{3}}{3A_{2}b}.$$

Based on Hooke’s law, the equivalent normal elastic coefficient $k_{s}$ of the beam can be calculated as,

(29) $$F=k_{s}w_{2}(l).$$

Hence, the equivalent normal elastic coefficient $k_{s}$ of the tactile sensor can be expressed as:

(30) $$k_{s}=\frac{1}{\frac{\left(2\alpha -\alpha^{2}\right)l^{3}}{2A_{1}b}+\frac{{\left(1-\alpha \right)}^{2}l^{3}}{3A_{2}b}}.$$

The relationship between the capacitance change and the deflection of the beam at x=l can be derived by plugging Equation (28) into Equation (13):

(31) $$\Delta C=\frac{C_{0}(z_{N1}-z_{3})}{\frac{\left(2\alpha -\alpha^{2}\right)l^{2}}{2}+\frac{{\left(1-\alpha \right)}^{2}A_{1}l^{2}}{3A_{2}}}w_{2}(l).$$

As seen from the Equations (11) and (12), the merit of the uniform-strength beam configuration is that the strain of the capacitive sensing sheet along the x-direction is uniform, and does not change with the coordinate points in the x direction. According to Equations (13) and (31), the capacitance change is linear to the force/deflection at the free end of the beam. Theoretically, the proposed sensor can be used to measure force and displacement.

## 3. Fabrication and Experimental Test

To verify the accuracy of the theoretical model, relationships between capacitance changes and the forces/displacements applied to the free end of uniform-strength cantilever beams were experimentally investigated. Based on these experiments, the sensing characteristics of sensors were studies in the quasi-static state.

### 3.1. Fabrication

To prove the accuracy of the theoretical model, we prepared three sensors based on three silicone rubber beams (uniform-strength cantilever beams) with different sizes and elastic moduli as listed in Table 1. The capacitive sensing sheets attached to the silicone rubber beams constitute a layer of dielectric (silicone rubber film) and two layers of ionic conductors. The polyacrylamide hydrogels containing lithium chloride [32] were used as ionic conductors, with an excellent water retention capacity.

#### 3.1.1. Synthesis of Ionic Conductors

The preparation process of the ionic hydrogel conductors is as follows [32]—(1) acylamide monomer (AM) and salt powder (LiCl·H_2_O) were dissolved in deionized water. The concentration of AM and LiCl·H_2_O was 2.2 and 8 mol/L, respectively. (2) A crosslinking agent (N, N’-methylenebisacrylamide, MBAA), a thermo-initiator (ammonium persulphate, AP), and an accelerator (N,N,N’,N’-tetramethylethylenediamine, TEMED) were added to the mixed solution. The amounts of MBAA, AP, and TEMED used were 0.06%, 0.17%, and 0.05%, respectively, of the AM weight. (3) The solution was transferred into a self-made glass mould and gelled in a drying oven at 50 ℃ for 2 h. (4) The prepared polyacrylamide hydrogel was placed in the chamber (25 ℃, 16% RH) for ≥120 h, then was used as the ionic conductor. The thickness and elasticity modulus of transparent and stretchable hydrogel are 0.462 mm and 1.8 kPa [33], respectively.

#### 3.1.2. Preparation of Dielectric

The material of the dielectric is a commercial silicone rubber (Eco-flex 0050, Smooth-On, USA). First, Eco-Flex prepolymers A and B were mixed in a 1:1 weight ratio, and the mixture was degassed. Subsequently, the mixture was casted onto polyethylene terephthalate (PET) films by using a laboratory casting equipment (MSK-AFA-L800, Hefei Ke Jing Materials Technology Co. Ltd., Hefei, China) and was cured at room temperature for 3 h.

#### 3.1.3. Preparation of Uniform-Strength Cantilever Beam

The uniform-strength cantilever beam is comprised of silicone rubber. Two types of silicone rubbers were used for the beams of the three sensors. One of the beams was fabricated using the silicon rubber (Eco-flex 0050), which was obtained by curing a mixed and degassed two-part Eco-Flex prepolymer in a 1:1 weight ratio for 3 h at room temperature. The Eco-flex used for the beam was curded into a silicon rubber sheet in the self-made glass mould. The elasticity modulus of Eco-flex 0050 is 83 kPa [34]. The other two beams were manufactured using the silicon rubber sheet (gjb001, DaoGuan, Shanghai, China). The elasticity modulus of the silicon rubber (gjb001) is 2.654 MPa (see Figure S2 of Supplementary Information).

The silicon rubber sheets as the beam, the dielectrics, and the ionic hydrogel conductors were cut into the aforementioned required shapes (see Figure 1) by using a laser cutting system (Versa Laser VLS2.30, Universal Laser Systems, Scottsdale, AZ, USA). The ionic conductor as the bottom electrode, the dielectric separated from the PET, and the ionic conductor as the top electrode, were then installed on the surface of the uniform strength beam by using the viscidity of the ionic conductors (polyacrylamide hydrogels).

**Figure 5:**
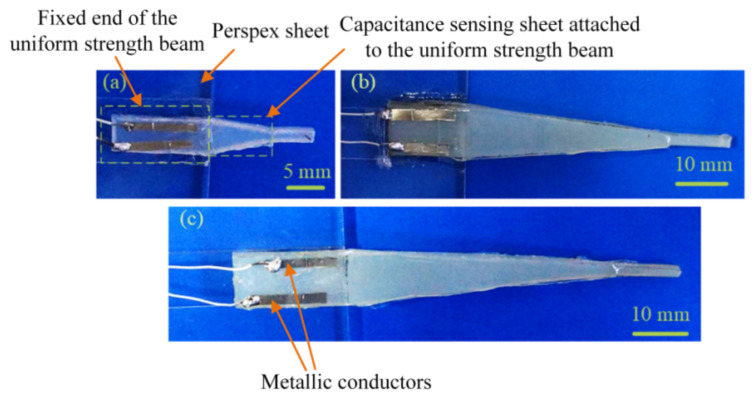
Tactile sensor samples, where (**a**) is No.1 sensor, (**b**) is No.2 sensor, and (**c**) is No.3 sensor.

The extremity of the uniform strength beam was pasted on a perspex sheet by employing a double-sided tape (VHB4910, 3M, Paul, MN, USA) and cyanoacrylate adhesive (502, Deli, Ningbo, China). Two metallic conductors (nickel plates) were fixed at the extremity for connecting ionic conductors and wires. Figure 5 illustrates the prepared sensors based on the uniform-strength cantilever beam. The capacitance-sensing sheets are almost transparent because the ionic conductors and dielectrics have excellent transparency.

### 3.2. Experimental Setup

**Figure 6:**
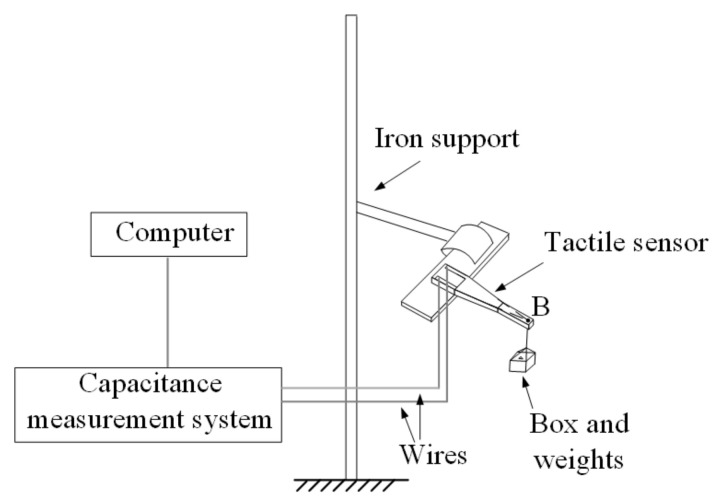
Schematic diagram of experimental setup for the force sensing analysis.

For the force sensing analysis, the perspex sheet (the extremity of the tactile sensor) was clamped to an iron support, as shown in Figure 6. To concentrate the load, a self-made paper box (10 mgf) and weights (F1, Penglai Shui Ling weight factory, Penglai, Shandong, China) were applied to the free end (point B) of the uniform-strength cantilever beam. We applied the concentrated force from 0 to 1.47 mN (150 mgf) by employing a constant load of 0.098 mN (10 mgf) for each step.

**Figure 7:**
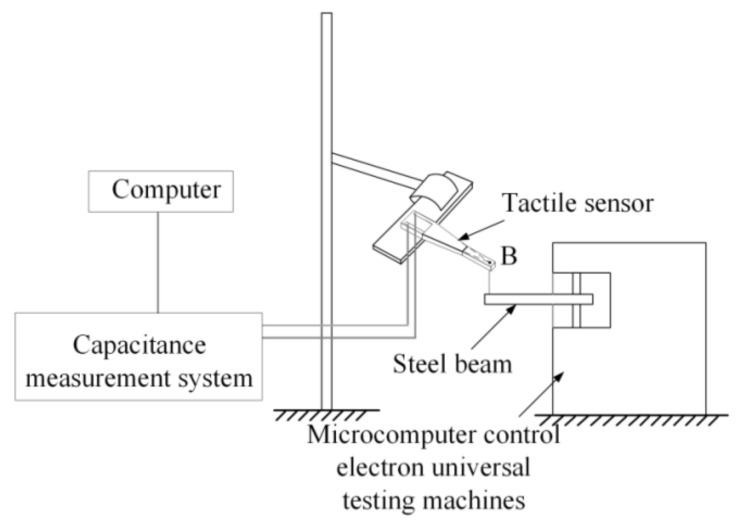
Schematic diagram of experimental setup for the displacement sensing analysis.

For the displacement-sensing analysis, the extremity of the tactile sensor also was clamped to an iron support (see Figure 7). To apply the displacement load, the free end (point B) is connected through an inextensible nylon wire to a steel beam fixed to microcomputer control electron universal testing machines (WDW-100C, Shanghai hualong test instrument co., Ltd., Shanghai, China). The displacement was achieved by applying vertical down tension to the nylon wire with a velocity and data acquisition frequency of 6 mm/min and of 20 Hz, respectively.

The capacitances of the tactile sensors were measured using a capacitance measurement system (PCapØ1 EVA-KIT, ACAM GmbH, Stutensee, Germany). The data acquisition frequency was 10 Hz. The measured initial capacitances of the No.1, No.2 and No.3 sensor were approximately 51.44, 169.09 and 201.51 pF, respectively.

### 3.3. Measurement

#### 3.3.1. Force vs. Capacitance Change

**Figure 8:**
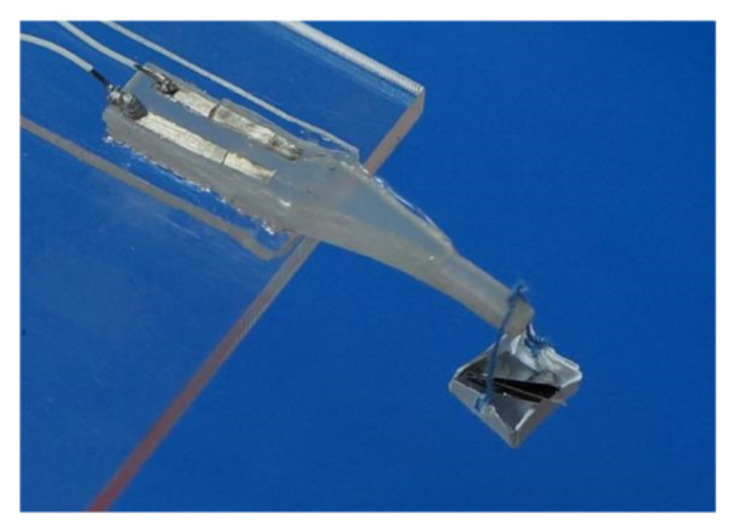
Photo of the force applied to the tactile sensor.

**Figure 9:**
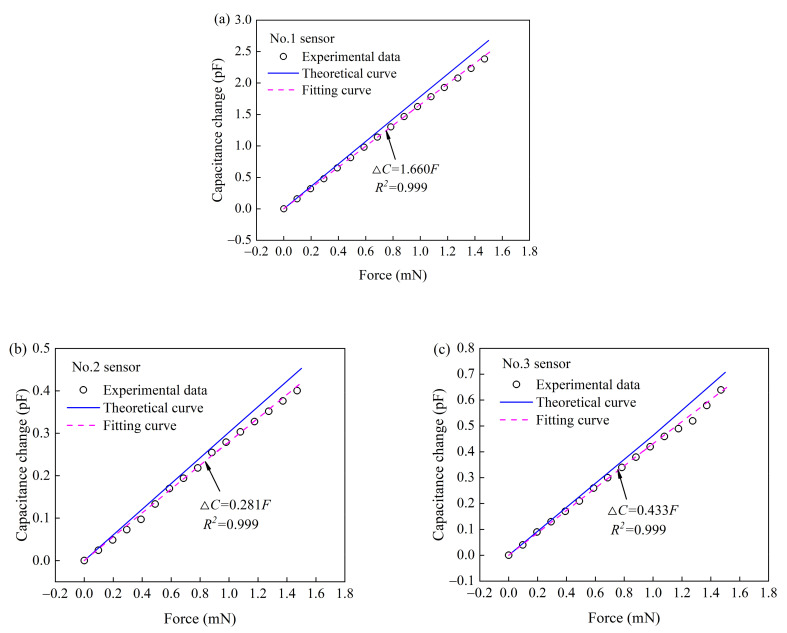
Capacitance change with the force applied to (**a**) No.1, (**b**) No.2, and (**c**) No.3 sensor.

To investigate sensing performances (capacitance change vs. force) of the tactile sensors, the force (box and weights) was applied to the free end of each uniform-strength cantilever beam, as shown in Figure 8. A good linear relationship was observed between the capacitance change and force in Figure 9. The slope of the fitting curve obtained from the experimental data (capacitance change vs. force) is the sensitivity of each tactile sensor (force measurement). The sensitivities (No.1, No.2 and No.3 sensor) are 1.660, 0.281, and 0.433 mN·pF^−1^, respectively. The theoretical sensitivities of the three tactile sensors are in a good agreement with the experiment results with errors 8.7%, 9.2%, and 10.7%, respectively.

#### 3.3.2. Displacement vs. Capacitance Change

**Figure 10:**
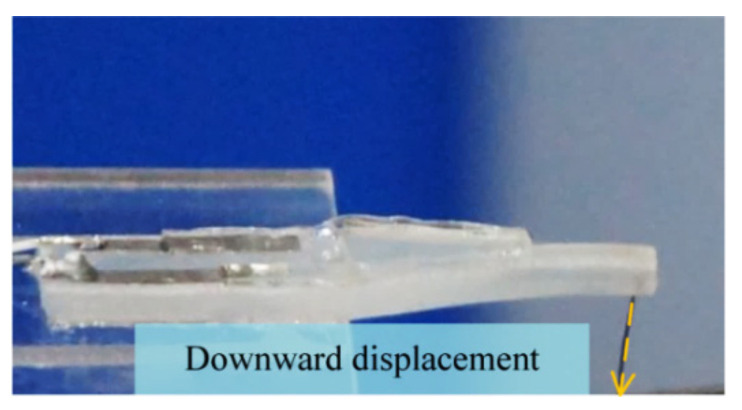
Photo of displacement applied to the tactile sensor.

**Figure 11:**
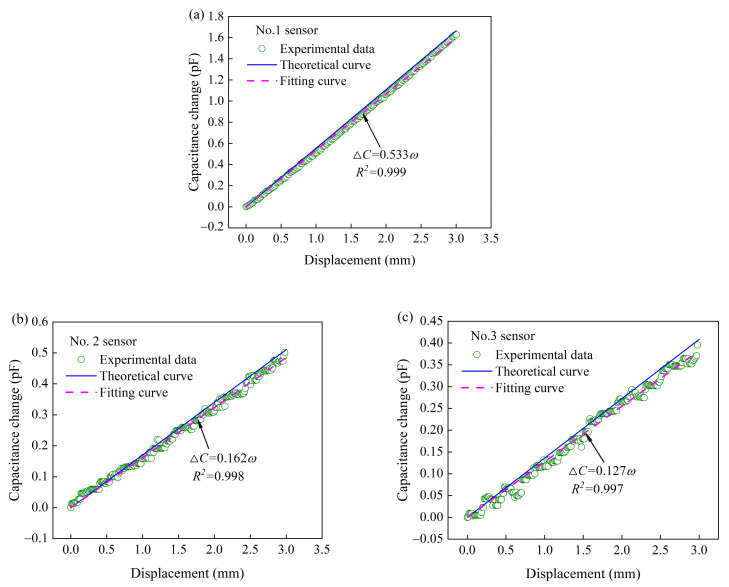
Capacitance change with the displacement applied to (**a**) No.1, (**b**) No.2, and (**c**) No.3 sensor.

To investigate sensing performances (capacitance change vs. displacement) of each tactile sensor (No.1, No.2 and No.3 Sensor), the displacement was applied vertically downward to the free end of each uniform-strength cantilever beam, as shown in Figure 10. The experimental results are presented in Figure 11. Each tactile sensor responds to an increase in capacitance with an increase in the displacement (0–3 mm). There is a good agreement between the theoretical predictions and the experimental results (No.1, No.2 and No.3 Sensor) with an error of 3.9%, 5.5%, and 7.1%, respectively. The relationships between displacements and capacitance changes are almost linear. The slopes of the fitting curves (capacitance change vs. displacement) of the tactile sensors (No.1, No.2 and No.3 Sensor) are 0.533, 0.162, and 0.127, respectively. That is, the sensitivities (displacement measurement) of the tactile sensors are 0.533, 0.162, and 0.127 pF·mm^−1^.

The No.1 sensor shows the highest sensitivity among the three sensors. Moreover, the No.1 sensor has an excellent linearity (R^2^ = 0.999), compared with the flexible pressure sensor with high linearity (R^2^ = 0.995) in the literature [21] and soft cantilever sensors in the literature [28,29,30].

## 4. Comparative Verification

In order to prove the superior characteristics (about sensitivity and softness) of the proposed sensor, its detection limit and equivalent stiffness (No.1 sensor) is comprehensively compared with those from current references.

**Figure 12:**
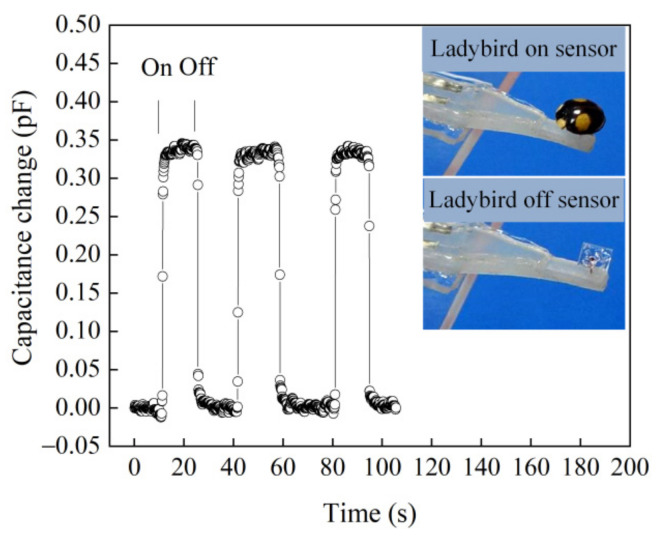
Capacitance change with time for placing on and off a dried ladybird (21 mgf).

**Figure 13:**
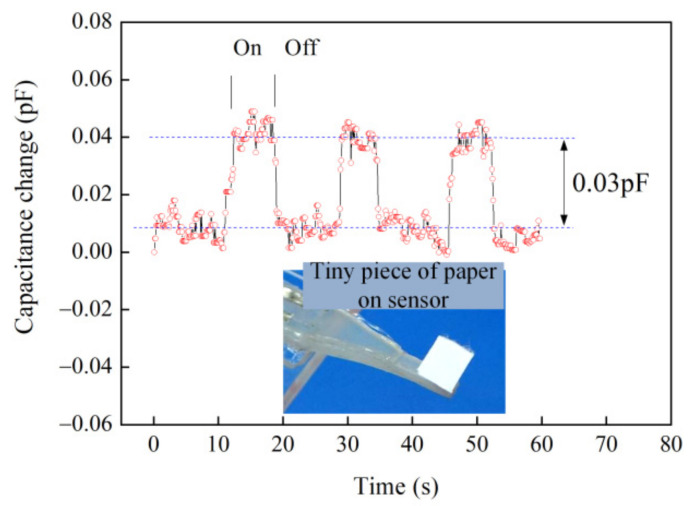
Capacitance change with time for placing on and off a tiny piece of paper (2 mgf).

First, several tests were conducted for analyzing the low detection limit of the tactile sensor (No.1 sensor). The sensor was reliably used to detect the ultra-small weights, such as a dried ladybird (weight—21 mgf) and a tiny piece of paper (weight—2 mgf). They were placed and removed on a small PET plate (3 mm^2^ × 3 mm^2^) fixed to the free end of the beam, as shown in Figure 12 and Figure 13. The ultra-small weights (21 and 2 mgf) caused obvious capacitance changes, which corresponded to the average values of 0.34 and 0.03 pF. The sensor can be used to measure force as low as 19.6 μN (2 mgf), which is a tenth of the lowest touch of female fingertip [35].

To compare with the flexible capacitive and resistive pressure sensors in the literatures, the sensitivity of the No.1 sensor was discussed for pressure measurement. According to the performances test of force sensing in Section 3.3.1, the fitting relationship between the capacitance change (ΔC) and the force (*F*) was ΔC=1.660F. The relative capacitance change was defined as $\Delta C/C_{0}$. The initial capacitance ($C_{0}$) of the No.1 sensor is 51.44 pF. Based on this, the fitting relationship between the relative capacitance change and force was $\Delta C/C_{0}=0.0323F$. The force (*F*) in the above equation can be converted into pressure (*P*), that is equal to *F* divided by the area of the PET plate (3 mm^2^ × 3 mm^2^) fixed to the free end of the beam. Hence, the relationship between the relative capacitance change and the pressure was $\Delta C/C_{0}=0.290P$. The sensitivity of No.1 sensor was 0.290 kPa^−1^ for pressure measurement. These results make our tactile sensor more sensitive than some flexible capacitive and resistive pressure sensors, as shown in Table 2.

**Figure 14:**
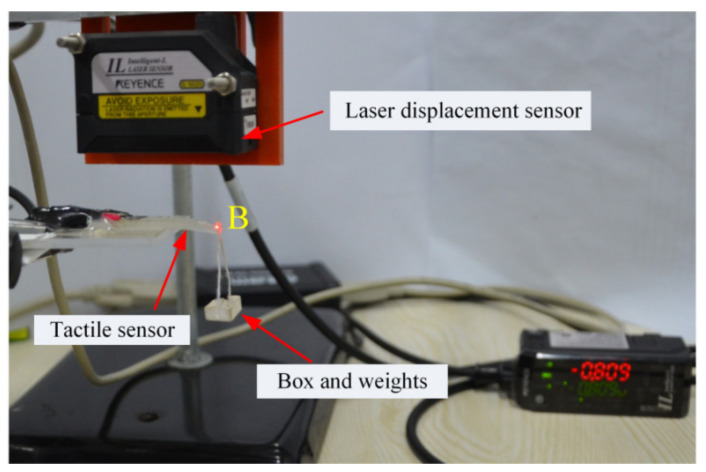
Experimental setup for the equivalent stiffness of the tactile sensor.

**Figure 15:**
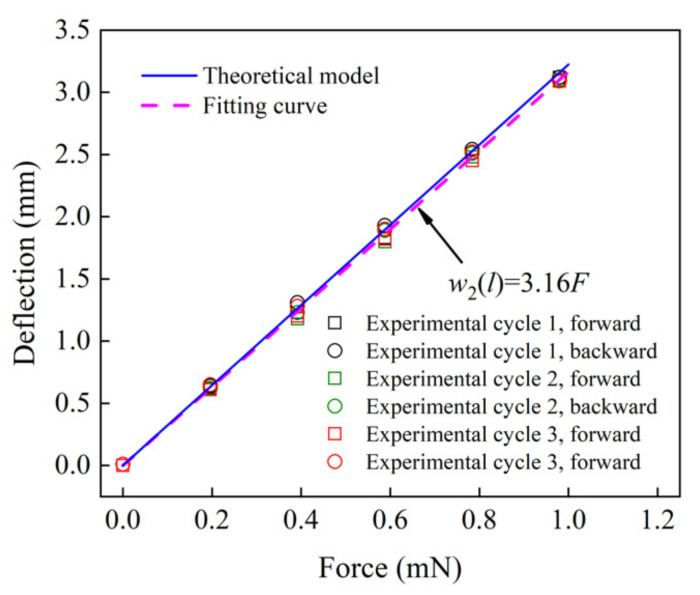
The deflection with the force applied at the free end of the tactile sensor.

Then, for the softness (equivalent stiffness) analysis of the proposed senor (No.1 sensor), the relationship between the force (*F*) and deflection ($w_{2}(l)$) at the free end of the uniform-strength cantilever beam is examined. The concentrated forces were applied to the free end of the beam, and the deflections of the free end (point B) were measured using an intelligent-L laser senor (lL-S025, KEYENCE Corporation, Osaka, Japan) (see Figure 14). The forces were loaded (0–0.98 mN) and unloaded (0.98–0 mN) at the free end three times. An increase in the deflection of the beam corresponded to an increase in the force at the free end in load–unload cycles, with a high consistency between the theoretical prediction and the experimental data (see Figure 15). Within the 0–0.98 mN measurement range, the relationship between the force and deflection was almost linear. The slopes of the curves experimentally and the theoretically obtained were 3.16 and 3.22 mm·mN^−1^, respectively, with an error of 1.7%. Hence, the equivalent stiffness (elasticity coefficient) of the tactile sensor was 0.31 N·m^−1^. Oleg et al. [48] reported the equivalent stiffness values of fat, skin and muscle of 83, 331, and 497 N·m^−1^, respectively. Therefore, the equivalent stiffness of the proposed sensor is considerably low, which is 0.4% and 0.1% of equivalent stiffness of fat and skin, respectively.

**Figure 16:**
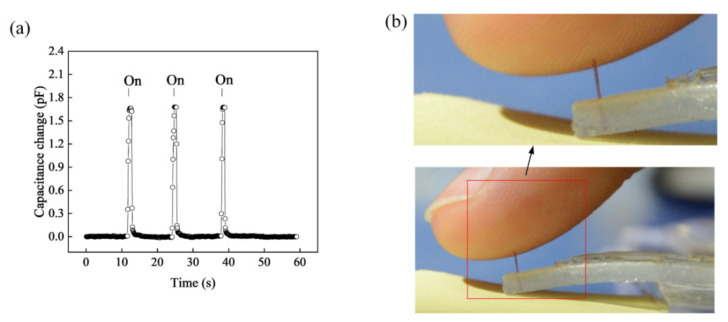
(**a**) Capacitance change with time for (**b**) fingertip pressing on and off the free end of the tactile sensor.

To further confirm the softness of the No.1 sensor, the sensor was employed to monitor fingertip movement. As an index fingertip pressed the needle at the free end of the beam of the tactile sensor three times, there were noticeable capacitance changes (see Figure 16a). The beam deflection at the free end induced by the pressure of the fingertip (fingertip movement) was approximately 3 mm each time, but the skin of the index finger was undeformed with the unaided eye (see Figure 16b). Because the human index finger is mainly composed of skin, fat, muscle, and bone, the equivalent stiffness of the index finger is higher than that of fat (83 N·m^−1^). Therefore, the equivalent stiffness of the sensor ($k_{s}$) is ≤0.4% (0.31 N·m^−1^ divided by 83 N·m^−1^) of the equivalent stiffness ($k_{F}$) of the fingertip. Because of the interactions between the index finger and the tactile sensor, the relationship $w_{F}/w_{2}(l)=k_{s}/k_{F}\leq 0.4\%$ was obtained, where $w_{2}(l)$ is the beam deflection at the free end, and $w_{F}$ is the index finger deformation. According to this equation, the index finger deformation is ≤0.4% of the beam deflection (approximately 3 mm). Therefore, the index finger remained nearly undeformed. This finding indicated that the tactile sensor can be used to high accurately monitor the fingertip movement with an error of ≤0.4%.

Hence, the results showed that the tactile sensor is extremely soft, and can be used to accurately measure the force of soft objects 20 μN–1 mN, which corresponds to the displacement of 70 μm–3 mm. It should be noted that the relationship between the force and displacement at the free end of the beam is based on $w_{2}\left(l\right)=3.16F$ as illustrated in Figure 16.

## 5. Conclusions

We constructed an extremely soft normal force/displacement sensor by using dielectric and ionic electrodes based on the cantilever beam of uniform strength. The theoretical model describing the relationship between the force/displacement and capacitance change is proposed. The theoretical predictions and the experimental results are in a good agreement. The experimental results demonstrate that the proposed sensor has great sensing characteristics, including excellent linearity (R^2^ = 0.999), high sensitivity of 0.533 pF·mm^−1^ and 1.66 pF·mN^−1^ for measuring the displacement and force, and the detection limit is as low as 70 μm and 20 μN, which is only 10% of the pressure threshold value of a female fingertip. Furthermore, the sensor is extremely soft, and its equivalent stiffness of 0.31 N·m^−1^ is 0.4% of that of fat. Those characteristics render it highly advantageous in measuring the displacement/force of soft objects. In addition, the sensor is light weight, simple to manufacture and inexpensive. This paper provides a new method for the measurement of the force/displacement of the flexible mechanism, which presents potential applications in many fields such as soft drives, soft robots, human motion, and health monitoring.

## Figures and Tables

**Table 1:** Parameters of the uniform strength beam of the prepared tactile sensors.

Number	Length (*l*)/mm	Width (*b*)/mm	Thickness (*h*_4_)/mm	Elasticity Modulus (*E*_4_)/kPa	Length Ratio (α)
1	15	5	2.368	83	0.6
2	50	10	2.513	2653	0.8
3	60	10	2.408	2653	0.8

**Table 2:** Comparison of performance of the No.1 sensor with the other sensors from current references.

Refs	Types	Sensitivity	Minimum Limit of Detection
[36]	Capacitive	0.1 pF·g^−1^	10 mN
[37]	Capacitive	0.17 kPa^−1^	8 mN
[21]	Capacitive	–	300 mgf
[38]	Capacitive	0.12 kPa^−1^	50 mgf
[19]	Capacitive	0.863 kPa^–1^	10 mgf
[9]	Capacitive	0.16 kPa^–1^	10 mgf
[39]	Capacitive	0.0049 kPa^−1^	1.9 mgf
[40]	Capacitive	193 kPa^−1^	0.3 mgf
[41]	Piezoresistive	0.19 kPa^−1^	93 mgf
[42]	Piezoresistive	1036.04 kPa^–1^	62 mgf
[43]	Piezoresistive	83.9 kPa^−1^	20 mgf
[44]	Piezoresistive	128.29 kPa^−1^	15 mgf
[45]	Piezoresistive	4.4 kPa^−1^	13 mgf
[46]	Piezoresistive	0.014 kPa^−1^	3 mgf
[3]	Piezoresistive	0.55 kPa^−1^	2.3 mgf
[47]	Piezoresistive	0.21 kPa^−1^	1.6 mgf
[35]	Touch of fingertip (Male)	–	0.55 mN
[35]	Touch of fingertip (Female)	–	0.19 mN
This work	Capacitive	0.29 kPa^−1^	2 mgf (0.0196 mN)

## Data Availability

The data in this paper are available on request from the corresponding author.

## Supplementary Materials

The following are available online at https://www.mdpi.com/article/10.3390/ma14071743/s1, Figure S1: Specimens of silicone rubber (gjb001) for uniaxial tensile test, Figure S2: Nominal stress vs. strain curve of the silicone rubber sheet (gjb001).
