# Supplementary material for: Extra-Soft Tactile Sensor for Sensitive Force/Displacement Measurement with High Linearity Based on a Uniform Strength Beam
Source: Materials (Basel). 2021 Apr 1;14(7):1743. doi: 10.3390/ma14071743 (PMC8037311; doi:10.3390/ma14071743)
Supplement: Supplementary file 1 [file materials-14-01743-s001.pdf]

materials

MDPI

Supplementary Material

Extra-Soft Tactile Sensor for Sensitive Force/Displacement Measurement with High Linearity Based on a Uniform Strength Beam

Na Ni [1], Xiaomin Xue [2,*] and Dongbo Li [1,*]

[1] School of Science, Xi'an University of Architecture and Technology, Xi'an 710055, China; nina@xauat.edu.cn (N.N.); ldb@xauat.edu.cn (D.L.)
[2] Department of Civil Engineering, Xi'an Jiaotong University, Xi'an 710054, China
* Correspondence: xuexm@mail.xjtu.edu.cn (X.X.); ldb@xauat.edu.cn (D.L.); Tel.: +86-17392778635 (D.L.)

Citation: Ni, N.; Xue, X.; Li, D. Extra-Soft Tactile Sensor for Sensitive Force/Displacement Measurement with High Linearity Based on a Uniform Strength Beam. *Materials* **2021**, *14*, 1743. https://doi.org/10.3390/ma14071743

Academic Editor: Mikhael Bechelany

Received: 8 February 2021
Accepted: 30 March 2021
Published: 1 April 2021

Publisher's Note: MDPI stays neutral with regard to jurisdictional claims in published maps and institutional affiliations.

Copyright: © 2021 by the authors. Licensee MDPI, Basel, Switzerland. This article is an open access article distributed under the terms and conditions of the Creative Commons Attribution (CC BY) license (http://creativecommons.org/licenses/by/4.0/).

Elasticity modulus test for silicone rubber (gjb001, DaoGuan, China)

The silicone rubber sheet (gjb001, DaoGuan, China) was cut into the shape shown in Figure S1. Uniaxial tensile experiments were carried out by electronic universal testing machine (CMT6530， SANS， USA) for three specimens of the silicone rubber sheet (gjb001). The relationship between nominal strain and nominal stress is shown in the Figure S2. According the fitting curve, the elasticity modulus of the silicone rubber (gjb001) is 2.654MPa.

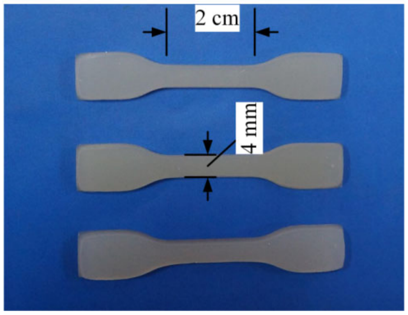

Figure S1. Specimens of silicone rubber (gjb001) for uniaxial tensile test.

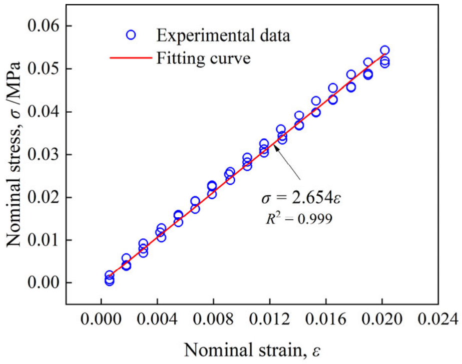

Figure S2. Nominal stress vs. strain curve of the silicone rubber sheet (gjb001).
